# The transcriptome sequencing and functional analysis of eyestalk ganglions in Chinese mitten crab (*Eriocheir sinensis*) treated with different photoperiods

**DOI:** 10.1371/journal.pone.0210414

**Published:** 2019-01-15

**Authors:** Yang-yang Pang, Cong Zhang, Min-jie Xu, Gen-yong Huang, Yong-xu Cheng, Xiao-zhen Yang

**Affiliations:** 1 National Demonstration Center for Experimental Fisheries Science Education, Shanghai Ocean University, Shanghai, China; 2 Key Laboratory of Freshwater Aquatic Genetic Resources, Ministry of Agriculture, Shanghai Ocean University, Shanghai, China; 3 Shanghai Engineering Research Center of Aquaculture, Shanghai Ocean University, Shanghai, China; Zhejiang University College of Life Sciences, CHINA

## Abstract

Photoperiod plays an important role in individual growth, development, and metabolism in crustaceans. The growth and reproduction of crabs are closely related to the photoperiod. However, as of yet, there are still no transcriptomic reports of eyestalk ganglions treated under different photoperiods in the Chinese mitten crab (*Eriocheir sinensis*), which is a benthonic crab with high commercial value in Asia. In this study, we collected the eyestalk ganglions of crabs that were reared under different photoperiods, including a control group (L: D = 12 h: 12 h, named CC), a constant light group (L: D = 24 h: 0 h, named LL) and a constant darkness group (L: D = 0 h: 24 h, named DD). RNA sequencing was performed on these tissues in order to examine the effects of different photoperiods. The total numbers of clean reads from the CC, LL and DD groups were 48,772,584 bp, 53,943,281 bp and 53,815,178 bp, respectively. After de novo assembly, 161,380 unigenes were obtained and were matched with different databases. The DEGs were significantly enriched in phototransduction and energy metabolism pathways. Results from RT-qPCR showed that TRP channel protein (TRP) in the phototransduction pathway had a significantly higher level of expression in LL and DD groups than in the CC group. We found that the downregulation of the pyruvate dehydrogenase complex (PDC) gene and the upregulation phosphoenolpyruvate carboxykinase (PPC) gene were involved in energy metabolism processes in LL or DD. In addition, we also found that the upregulation of the expression level of the genes Gαq, pyruvate kinase (PK), NADH peroxidase (NADH) and ATPase is involved in phototransduction and energy metabolism. These results may shed some light on the molecular mechanism underlying the effect of photoperiod in physiological activity of *E*. *sinensis*.

## Introduction

Photoperiod is important to individual growth, development, reproduction and energy metabolism [1–3]. Researchers have found that the survival was high when combined with longer photoperiod, and weight gain and specific growth rate were higher at shorter photoperiods of crayfish (*Astacus leptodactylus*) [4]. There were also low hatching rates and population numbers when calanoid copepods (*Acartia grani*) were kept under constant light conditions [5]. The photoperiod also affects molting and influences the sexual maturation of crustaceans [6–9]. For example, a long photoperiod can accelerate the rate of metamorphosis in the lobster (*Panulirus japonicus*)[10]. The effects of photoperiod on energy metabolism have also been reported in crustaceans. For example, the digestive enzymatic activity of the general proteases trypsin and chymotrypsin were affected by the photoperiod in the prawn (*Macrobrachium tenellum*) [7]. Similarly, reactive oxygen species and lipid peroxidation are regulated by the glutathione system, which is influenced by photoperiods in crayfish (*Procambarus clarkii*)[11]. In addition, changes in energy metabolism due to the photoperiod have been reported in adult krill (*Euphausia superba*) and Arctic copepod (*Calanus glacialis*) [12, 13]. However, the study of the effects of photoperiod on *E*. *sinensis*, which is an economically important aquaculture freshwater species in China, is still sparse.

Eyestalks are an important phototransduction organ that can receive light signals through photoreceptors [14–16]. Eyestalk ganglions, located in eyestalk, play an important role in phototransduction, energy metabolism, and endocrine regulation. A previous study showed that damaged eyestalk ganglion can lead to a destruction of the phototransduction pathway [17]. Moreover, some studies have found that eyestalks are involved in hatchability of embryos and gonadal development, such as in the crab (*Dyspanopeus sayi*) [18] and the mud crab (*Scylla paramamosain*)[19]. It was previously known that levels of the crustacean hyperglycemic hormone (CHH) in eyestalks are important for energy metabolism via the regulation glucose levels [20, 21], and these levels were controlled by photoperiods in the crayfish (*P*. *clarkii*) [22]. In our previous studies, we found that the eyestalks of *E*. *sinensis* express the DA2 receptor which participates in light adaptation during the dark hours [23]. However, there still lack information about the influence of photoperiods on the gene expression of eyestalk ganglion in *E*. *sinensis*.

The TRP channel protein (TRP) is a transient receptor potential cation channel with very diverse permeation and gating properties and participates in sensory and motile regulation process [24]. It is a vital gene in the phototransduction pathway [25]. Previous studies have shown that photoperiods can affect the TRP expression level in mice, TRP is involved in clock gene oscillations and energy balance control [26]. TRP can also change the osmolarity and fluid flow of individual cells in different environments [27]. For example, TRP can induce the increased levels of Ca^2+^, which further reduces the photoreceptor sensitivity of crayfish [28]. Additionally, both the pyruvate dehydrogenase complex (PDC) gene and the phosphoenolpyruvate carboxykinase (PPC) gene are related to energy metabolism [29, 30], and their activity can be influenced by photoperiods in mammals [31, 32].

The current study, the first to our knowledge, analyzes eyestalks ganglion transcriptomes of *E*. *sinensis* in all bright (LL), all dark (DD) and normal light (CC) conditions using Illumina HiSeq^TM^2000 technology. We attempt to identify important pathways and genes involved in photoperiod regulation. This study also provides a basis for further research.

## Materials and methods

### Experimental animals and sampling

Chinese mitten crabs (*E*. *sinensis*) were obtained from the Shuxin crab base in Chongming Island (121°30′~121°40′ E, 31°34′~31°37′ N), Shanghai (China) with body weights of 17.18 ± 2.2 g. They were stocked in clear glass aquaria (Length: Width: Height = 130: 60: 40 cm, Water depth = 25 cm) for 1 week, with a circulating system containing thoroughly aerated freshwater and UV-treated PVC tubes as shelter. The crabs were fed a basal diet once daily at 20:00 and were kept under a natural photoperiod of 12L: 12D. During the experiment, crabs were kept in a temperature of 19~22°C, a pH of 7.6~7.8, a dissolved oxygen concentration of at least 6.0 mg/L.

This study investigated whether different photoperiods affected the nervous system of *E*. *sinensis*. Crabs were exposed to one of three photoperiods including control (L: D = 12 h: 12 h, named CC), constant light (L: D = 24 h: 0 h, named LL) or constant darkness (L: D = 0 h: 24 h, named DD). Within the tank (Length: Width: Height = 37: 24.5: 11 cm) 10 crabs were randomly placed as a group, a light intensity of 100 lux was used, and treatments continued for 7 d. Then, crabs were frozen on ice, and the eyestalks were harvested and stored at -80°C until RNA isolation.

### RNA extracted, read alignment and RNA-seq analysis

Total RNA was extracted using RNAiso^TM^Plus (TaKaRa) according to manufacturer’s instructions. Qualified total RNA was further purified with a RNeasy micro kit (Cat#74004, QIAGEN, GmBH, Germany) and RNase-Free DNase Set (Cat#79254, QIAGEN, GmBH, Germany). The RNA concentration and purity were determined using a Nanodrop2000, and RNA quantity was measured by denaturing formaldehyde agarose gel electrophoresis to examine integrity and measured with an Agilent Bioanalyzer 2100 (Agilent Technologies). Only high-quality eyestalk RNA samples were used for cDNA synthesis, so in the end, there were two qualified samples in each group for cDNA synthesis.

The RNA integrity numbers (RINs) of samples was required to be more than 7.0 when RNA-seq transcriptome libraries were prepared. Poly-T oligo-linked magnetic beads were used to purify PolyA mRNA from total RNA, and the intact mRNA was broken into fragments with bead washing buffer and metal bath. Aforementioned mRNAs were used as templates to synthesize first-strand complementary DNA (cDNA). Then, second-strand cDNA was synthesized using Resuspension Buffer, EtOH and Buffer. Next, cDNA, was end-repaired, a base was added to the 3’ end, and the cDNA was amplified with PCR. Finally, the constructed cDNA libraries were sequenced with Illumina HiSeq^TM^2000.

### De novo assembly and annotation of the transcriptome

Raw reads from Illumina HiSeq^TM^2000 may contain sequencing primers and low-quality sequence, which can affect analytical quality. Therefore, the raw reads were cleaned through three steps: (a) linger sequences were discarded; (b) Q < 20 (Q = -10log_10_E) bases were removed; and (c) reads length shorter than 25 bp also discarded. Then, the clean reads were used for de novo assembly with Trinity (Minimum contig length> = 400 bp).

For annotation, the assembled final unigenes with screening condition (E-value<1e-5) were annotated using the NCBI protein nonredundant (Nr) and UniProt database. The top five unigenes that were compared with the CDD database by rpstblastn (http://www.biomedcentral.com/content/supplementary/1471-2105-13-42-s1/Cloud-BioLinux-Package-Documentation/docs/rpstblastn.html) were annotated using COG (Cluster of Orthologous Groups of proteins) classification. The unigenes were also classified with the GO (Gene Ontology) and KEGG (Kyoto Encyclopedia of Genes and Genomes) databases.

### Analysis of differentially expressed genes

The clean reads were mapped per sample to the corresponding gene for read counts. The expression levels of genes were determined by using Express software and the FPKM (fragments per kilobase of exon model per million mapped reads) method. Analysis of differential expression levels across samples was performed in edgeR, and significantly differentially expressed genes (DEGs) were calculated (q-value <= 0.05, Fold-change>=2). Significantly enriched terms were obtained by mapping DEGs to the corresponding GO term. Similarly, significantly enriched KEGG pathways were obtained.

### Quantitative real-time PCR verification

Three DEGs were randomly chosen for quantitative real-time PCR (RT-qPCR) to validate the accuracy of the RNA-seq results. The total RNA which was returned after sequencing (three samples in each group) was used as template to synthesize first-strand cDNA with PrimeScript RT Master Mix (Cat. No. RR036A, TaKaRa). Primers were designed based on the DEGs with Primer Premier 5.0 (Table 1).

**Table 1:** Primers were selected for RNA-seq validation by RT-qPCR. Three genes were named that TRP channel protein (TRP), pyruvate dehydrogenase complex (PDC), Phosphoenolpyruvate carboxykinase (PPC).

Gene	Forward primer (5’-3’)	Reverse primer (5’-3’)
18S	TCCAGTTCGCAGCTTCTTCTT	AACATCTAAGGGCATCACAGA
TRP	GTGTGGGTGTGACGAGTGCG	TCCTTGGAGGAGAGGGCGAT
PDC	CCGAGCGTCTTCATCTGCGA	ACACTGGTGCCCATGCCATA
PPC	CCAACAAAAACACCGTGTCG	GTTCCGCTGATTTACGAATC

The reaction system of RT-qPCR contained 5 μl of 2×SYBR Premix Ex TaqTM (TaKaRa, Japan), 1 μl of diluted first-strand cDNA, 3.4 μl of PCR-grade water, 0.2 μl of ROX Reference Dye II, and 0.2 μl of each primer [33]. The mixtures were run under the following conditions: 95°C for 30 s; followed by 40 cycles of 95°C for 5 s, and 60°C for 34 s; in addition, 95°C for 15 s, 60°C for 1 min, and 95°C for 15 s using the ABI 7500 Real-Time PCR System (Life Technology, USA). We used 18S ribosome RNA (18S) as a reference gene. The DEGs expression levels were calculated using the 2^-ΔΔCt^ method [23].

### Statistical analysis

The data were expressed as mean ± S.D. values, and One-way analysis of variance was used for comparisons among the groups. A *P* value <0.05 indicated statistically significant difference.

## Results

### Transcriptome sequencing and reads assembly

**Fig 1:**
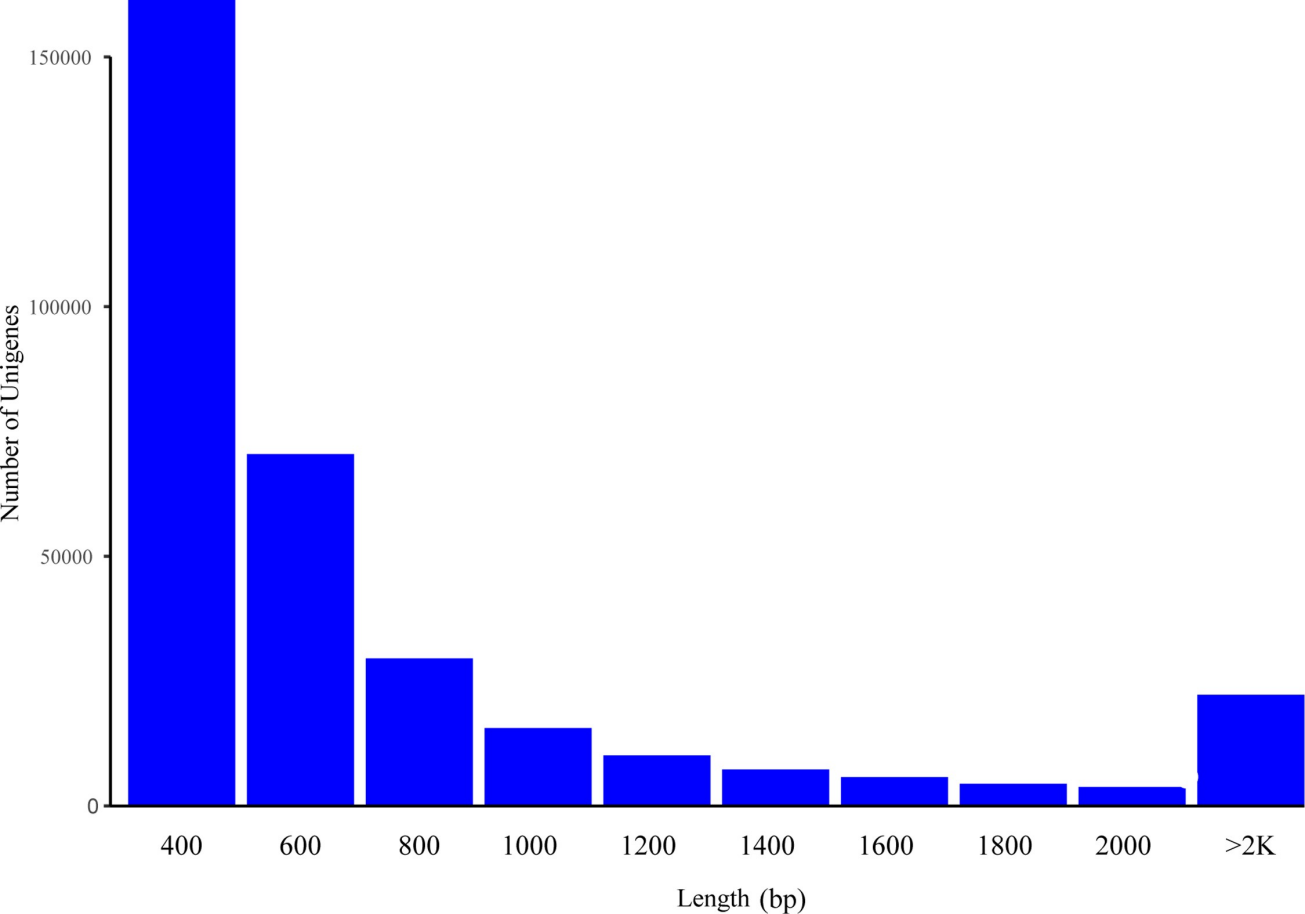
Unigenes distribution.

We obtained 51,786,884 bp raw reads from CC, 58,383,804 bp raw reads from LL, and 59,026,446bp raw reads from DD. Clean reads were selected by excluding reads that did not conform to the requirements. Thus, the clean reads of CC, LL and DD were 48,772,584 bp, 53,943,281bp and 53,815,178 bp, respectively, (Table 2) and they were used for de novo assembly. Using this, we obtained 330,686 unigenes that contained the 161,380 unigenes that were 200–400 bp, 70,473 unigenes that were 400-600bp and 22,260 unigenes that were more than 2000 bp in length (Fig 1). Unigenes were used for further functional analysis.

**Table 2:** Summary of the RNA-Seq reads production after quality trimming and adapter clipping of CC, DD and LL groups.

Sample	Clean reads	Clean ratio(%)	rRNA trimed	rRNA ratio(%)	GC(%)
CC	49,383,049	95.36	48,772,584	1.24	45
DD	55,481,901	95.03	53,943,281	2.77	47
LL	56,185,316	95.19	53,815,178	4.22	47

### Unigenes functional annotation

**Fig 2:**
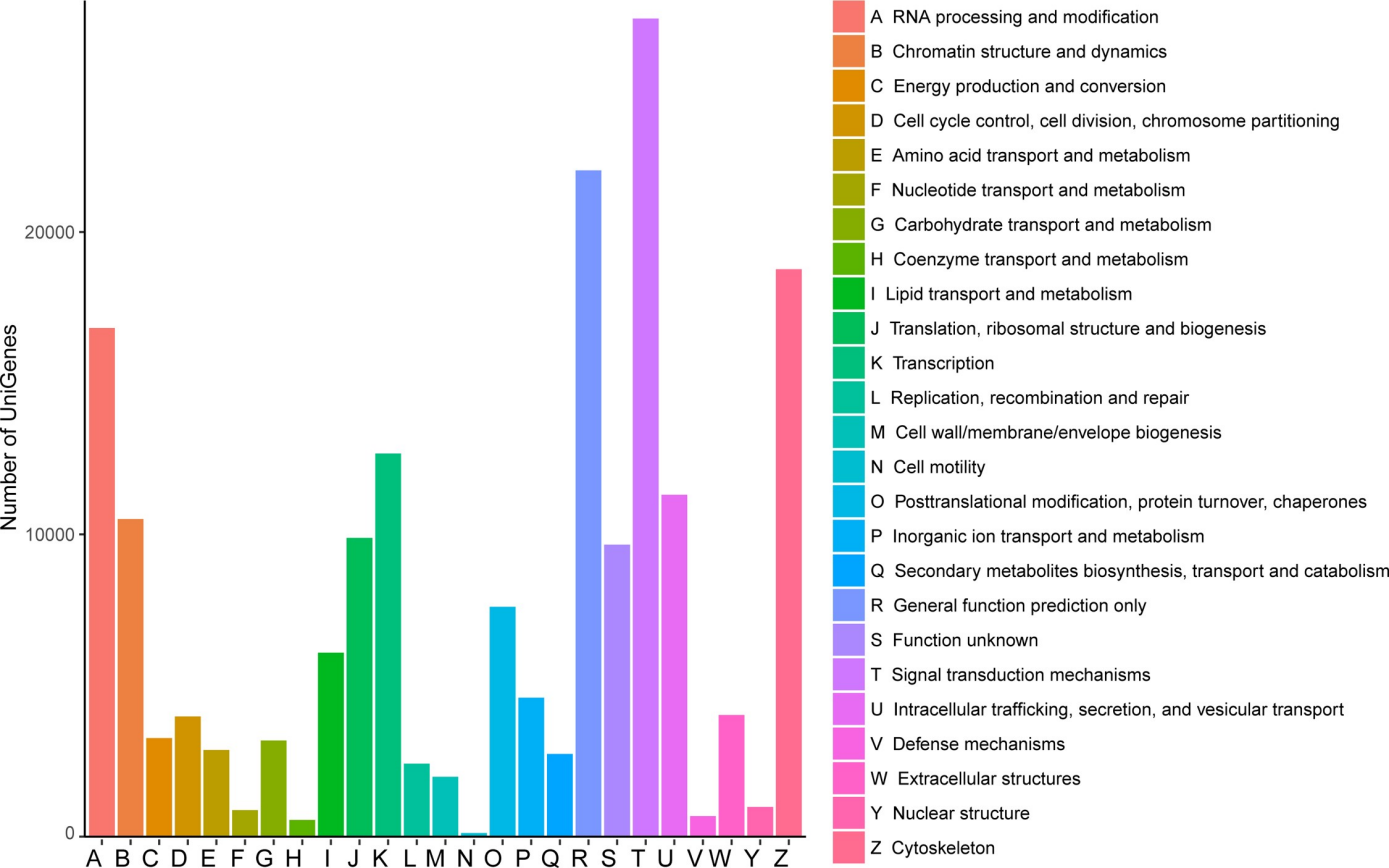
Unigene COG annotation.

COG classification was used to analyze unigenes function, and 184,664 unigenes were classified into 25 COG clusters, which were mainly enriched in signal transduction mechanisms (14.65%), general function prediction only (11.93%), cytoskeleton (10.16%), RNA processing and modification (9.11%) and transcription (6.86%) (Fig 2).

**Fig 3:**
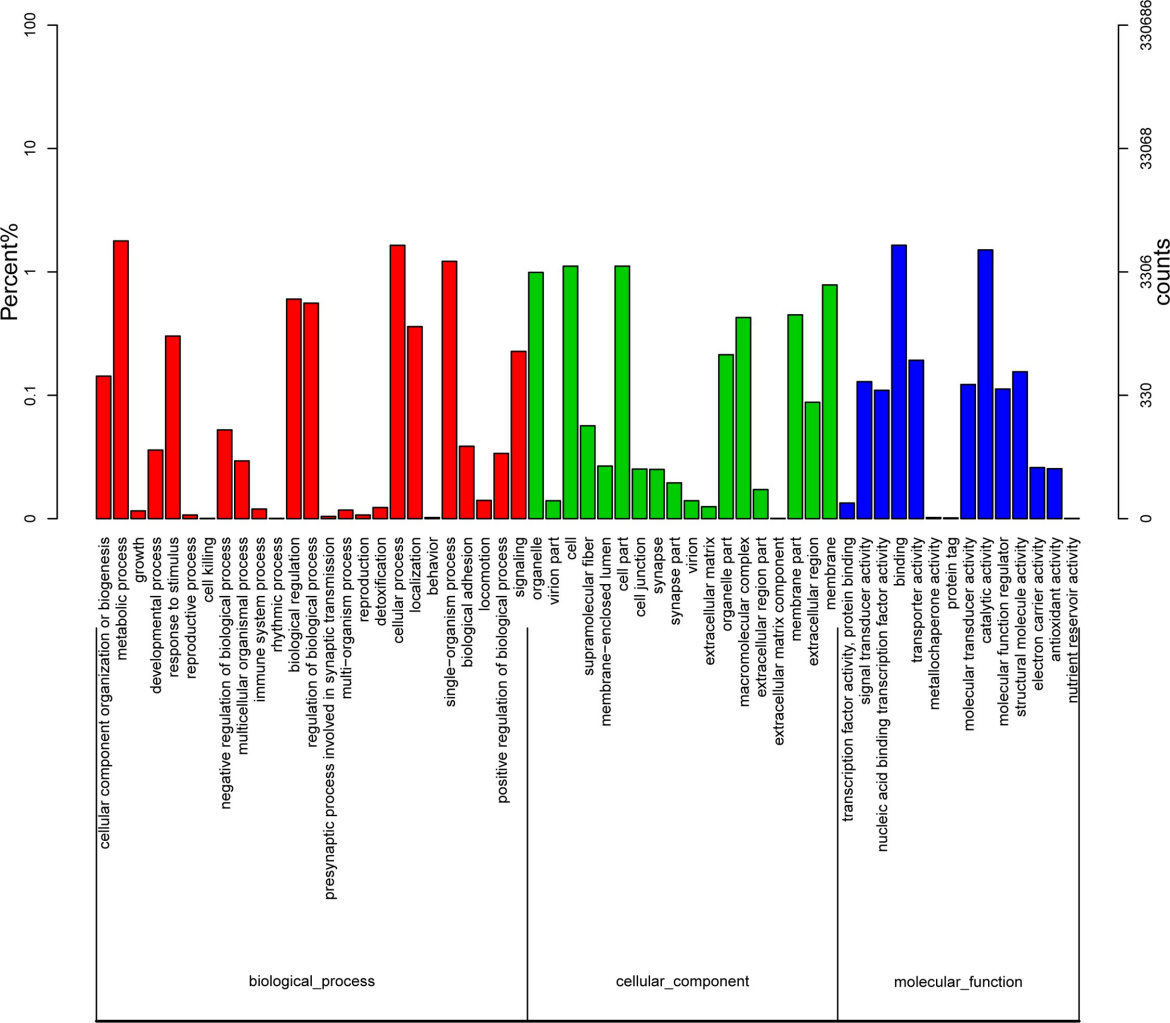
Unigene GO annotation.

A total of 74,804 unigenes were obtained by GO annotation, which were assigned to three groups: molecular function, cellular component and biological process, and further divided into 57 categories. Most of them were concentrated to metabolic process, organelle and catalytic activity, etc. (Fig 3).

**Fig 4:**
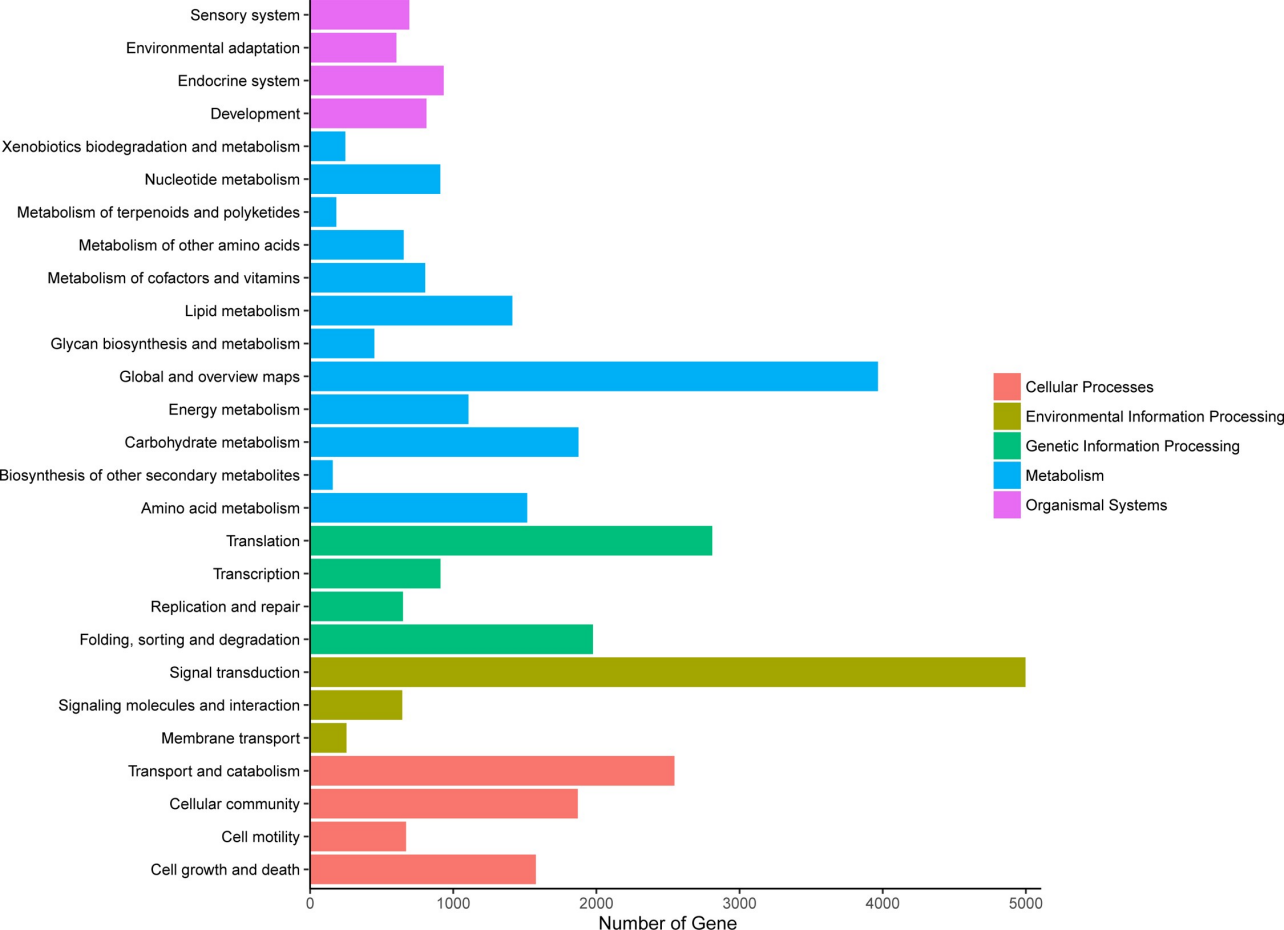
Unigene KEGG pathway annotation.

Finally, we analyzed the signaling pathways of unigenes with a KEGG KAAS online pathway analysis tool (http://www.kegg.jp/blastkoala/), a total of 3412 unigenes were mapped to five processes: metabolism (48.1%), environmental information processing (18.4%), genetic information processing (13.6%), cellular processes (13.1%), and organismal systems (6.8%). The maximum concentration of unigenes in metabolism was in the global and overview maps pathways, followed by carbohydrate metabolism, amino acid metabolism and lipid metabolism pathway. The pathways which contained the most unigenes of the other four processes were signal transduction, translation, transport and catabolism, and endocrine system, respectively (Fig 4).

### DEGs analysis

**Fig 5:**
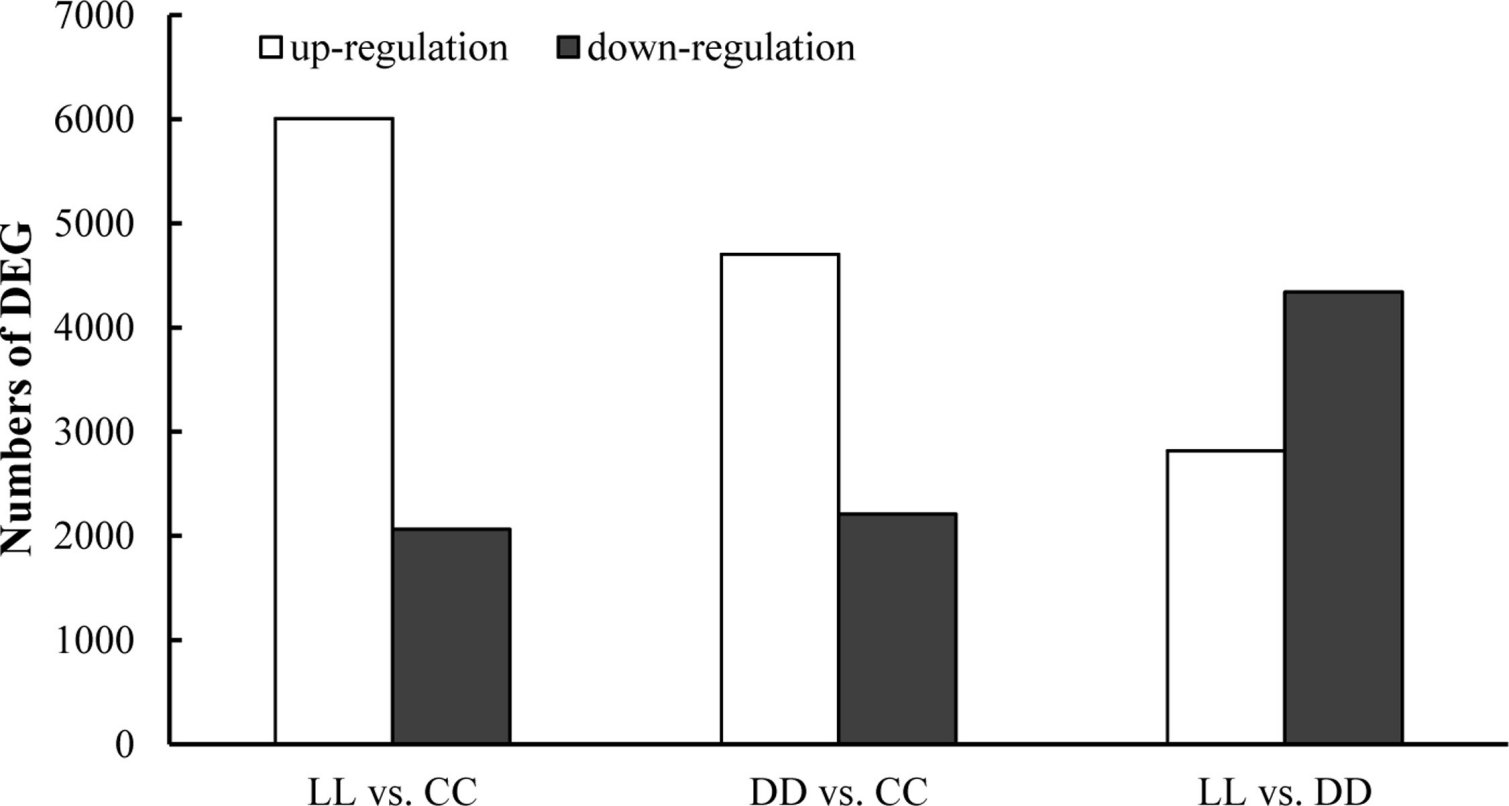
Numbers of DEGs in LL vs. CC, LL vs. DD, and DD vs. CC.

To analyze the DEGs among the different samples, we found that the number of up-regulated DEGs between the LL vs. CC was 6,008, and down-regulated DEGs were 2,064. The up-regulated DEGs in DD vs. CC were 4,704 and down-regulated DEGs were 2,210. Between the LL vs. DD, the up-regulated and down-regulated DEGs were 2,817 and 4,341, respectively (Fig 5).

**Fig 6:**
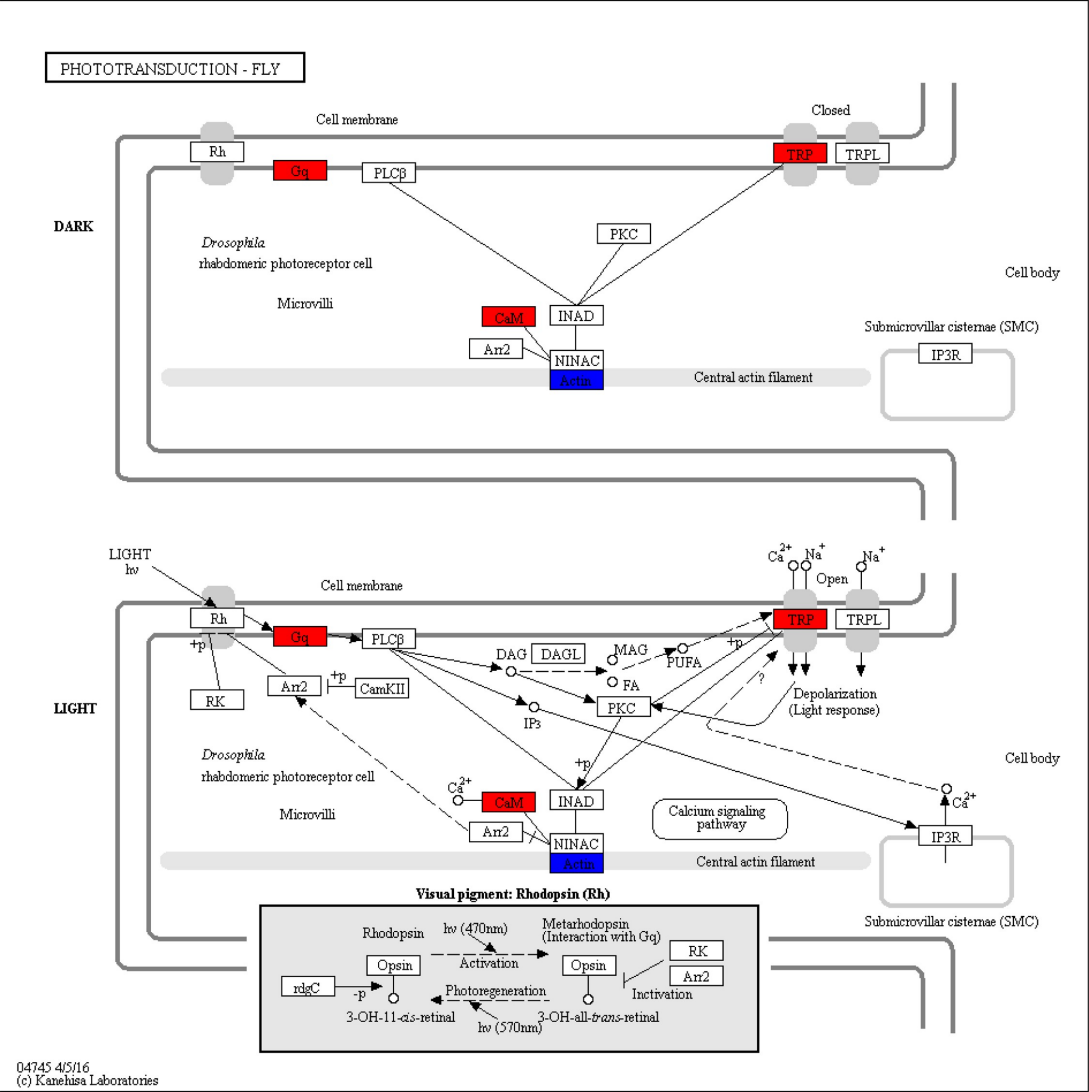
Phototransduction pathway (DD vs. CC).

**Fig 7:**
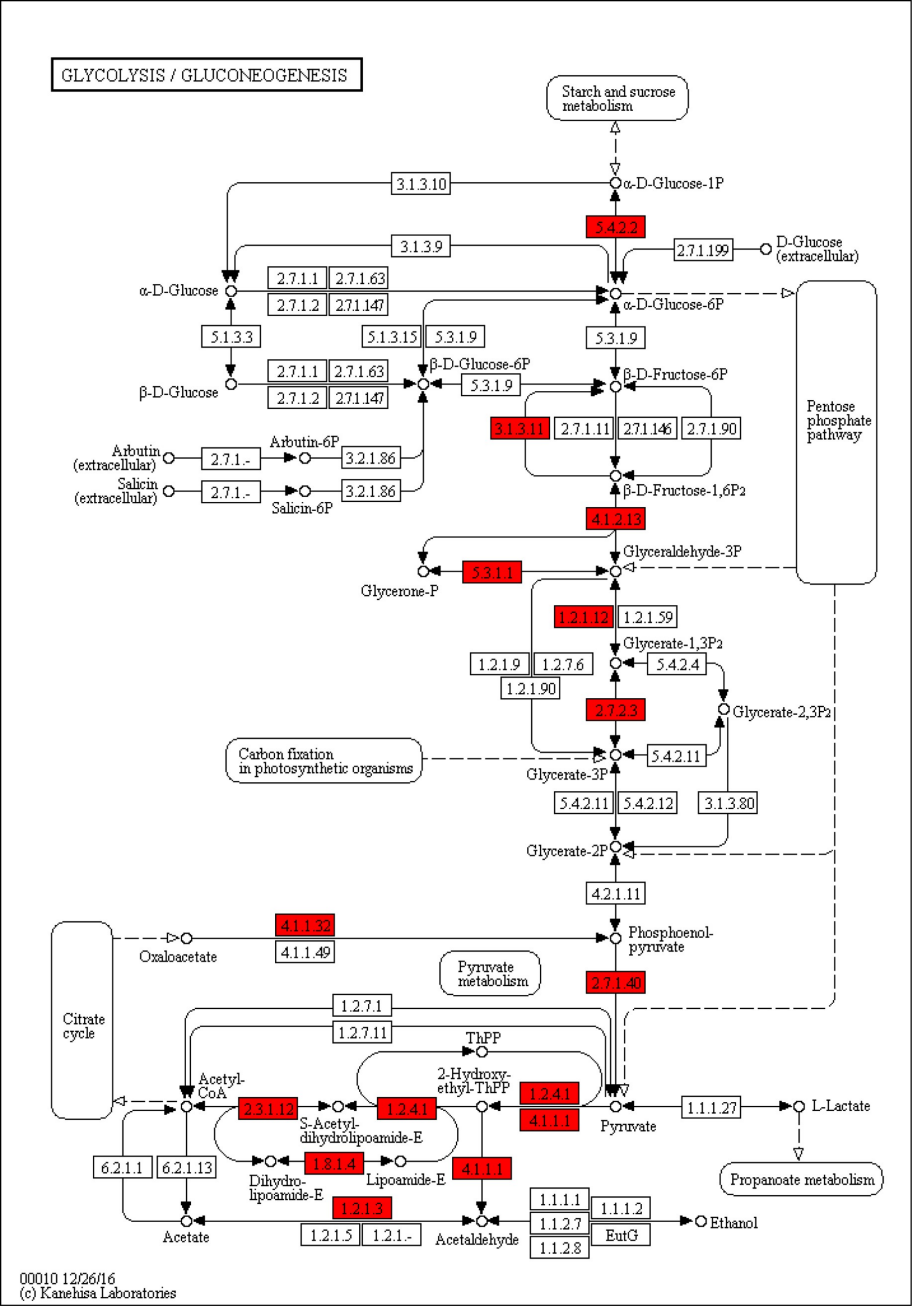
Glycolysis / Gluconeogenesis pathway (DD vs. CC).

**Fig 8:**
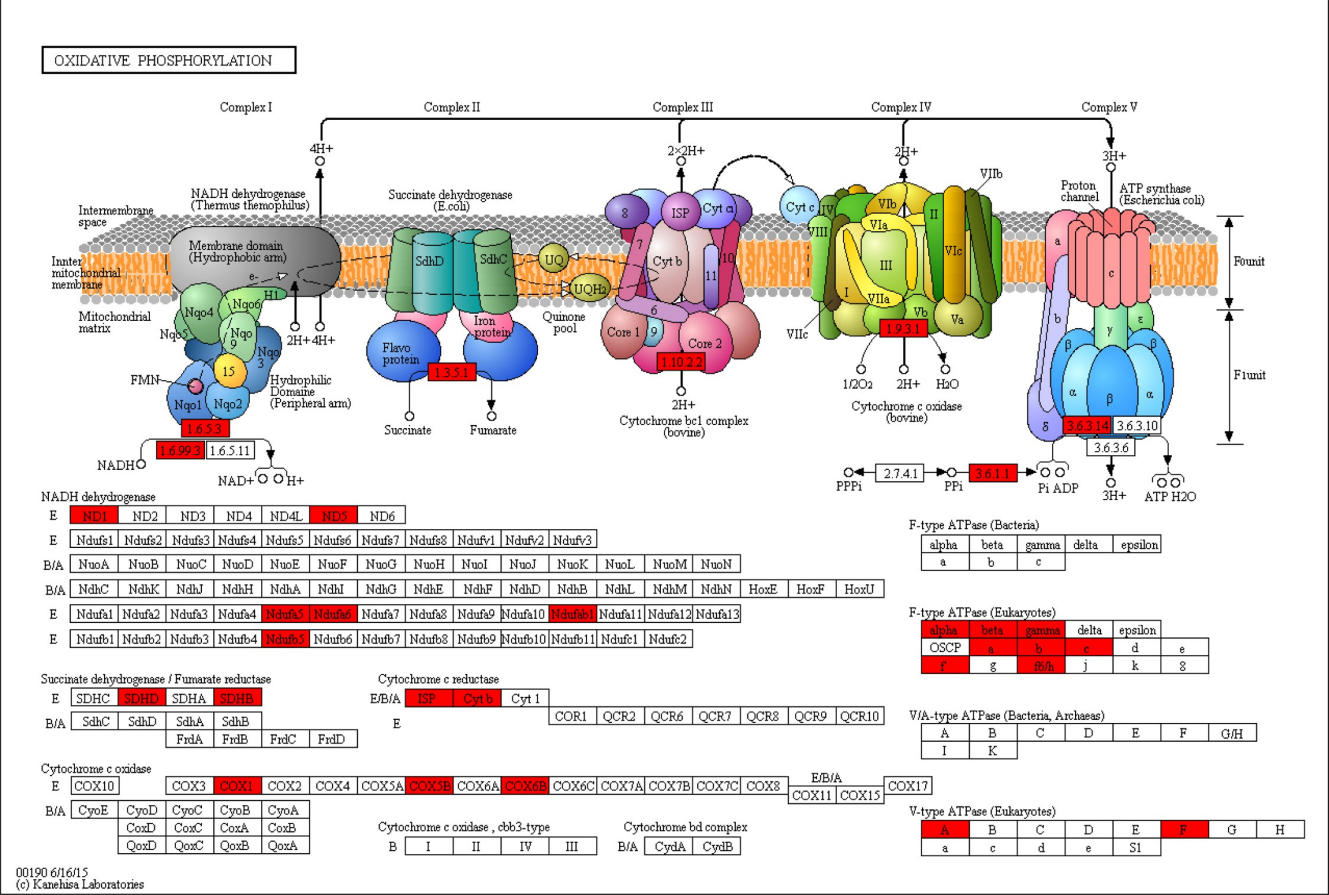
Oxidative phosphorylation pathway (LL vs. CC).

The significant DEGs were assigned to 196, 180 and 212 KEGG pathways for LL vs. CC, LL vs. DD and DD vs. CC, respectively. The significantly enriched pathways (q-value<0.05) are shown in Table 3. We found that the phototransduction (Fig 6) and glycolysis / gluconeogenesis (Fig 7) pathways were only included in the DD vs. CC comparison; oxidative phosphorylation (Fig 8) pathway was included only in the LL vs. CC comparison, but no significantly enriched pathway was found in the LL vs. DD comparison. We also found that the TRP has a significant upregulation in the phototransduction pathway. The PDC and PPC genes were found in glycolysis/ gluconeogenesis pathway, but PDC was significantly down-regulated and PPC was significantly up-regulated. Next, we obtained the significantly enriched GO terms from the DEGs with a *p*-value<0.05. There we had 23, 16 and 42 significantly enriched terms in LL vs. CC, LL vs. DD and DD vs. CC, respectively. The terms of ‘metabolic process’, ‘cell’, and ‘catalytic activity’ were the most highly enriched in the three groups (S1, S2 and S3 Figs). Some of the DEGs clustered in ‘developmental process’ and ‘growth’ in the LL vs. CC and DD vs. CC comparisons (S1 and S3 Figs).

**Table 3:** Results of KEGG Pathway of Chinese mitten crab kept in LL vs. CC, LL vs. DD and DD vs. CC.

pathway	DEGs with pathway			pathway ID
	LL vs. CC	DD vs. CC	LL vs. DD	
Ribosome	107	77	—	ko03010
Oxidative phosphorylation	38	37	—	ko00190
Phototransduction	—	21	—	ko04745
Glycolysis / Gluconeogenesis	—	25	—	ko00010
Carbon fixation in photosynthetic organisms	12	14	—	ko00710
Fluid shear stress and atherosclerosis	28	30	—	ko05418
Cutin, suberine and wax biosynthesis	—	2	—	ko00073
Phagosome	38	—	—	ko04145
Apoptosis	30	—	—	ko04210

### RT-qPCR identified DEGs expression

**Fig 9:**
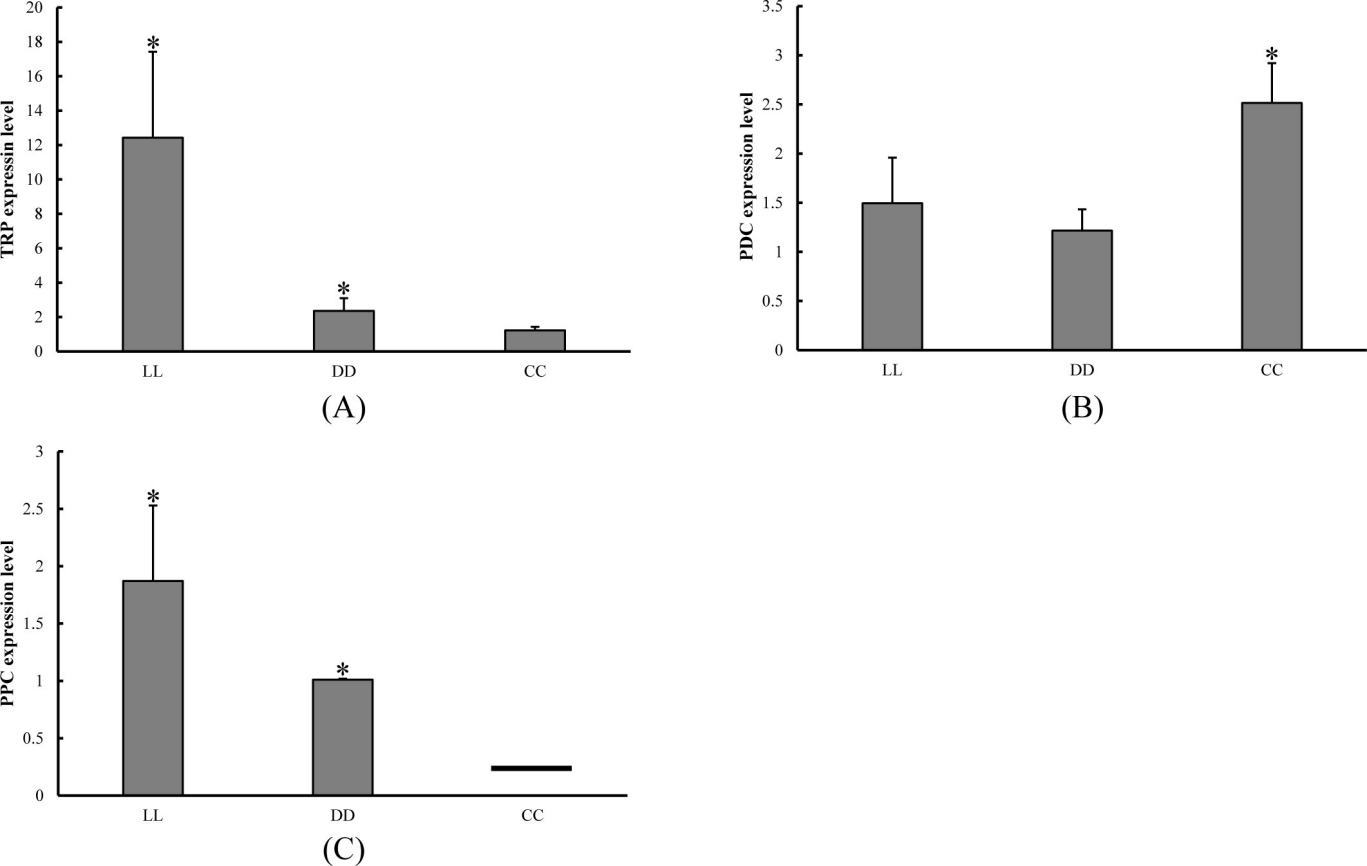
RT-qPCR analysis of 3 selected DEGs. Relative expression levels are shown for (A) TRP; (B) PDC; (C) PPC. Asterisks above the bars indicates significant differences (*P* < 0.05) between control (CC) and LL or DD group. ——indicates that PPC had no expression in CC group.

Three DEGs were selected to verify the RNA-Seq results. The results of RT-qPCR confirmed that TRP was significantly up-regulated in the constant light and constant darkness groups when compared with the control group (Fig 9A). Additionally, PDC was significantly down-regulated in the LL and DD groups (Fig 9B). We also found that PPC had no expression in CC group, but significant expression levels in the LL and DD groups, confirming the results from the RNA-Seq (Fig 9C).

## Discussion

This is the first report on the transcriptome of eyestalks ganglion in *E*. *sinensis* that were obtained with Illumina HiSeqTM2000 after exposure to different photoperiods. From the results we obtained, unigenes found to be primarily enriched in signal transduction mechanisms and metabolism with COG, GO and KEGG annotation analysis These results indicate that photoperiod conditions can also affect signal transduction and energy metabolism in *E*. *sinensis* as in other animals, such as *E*. *superba*, *C*. *glacialis* and *P*. *clarkii* [12, 13, 22].

Further analysis of DEGs, we found that the phototransduction pathway was significantly enriched in DEGs (Fig 6). In the phototransduction pathway, the gene TRP has a significant upregulation (Fig 6A) in LL vs. CC and in DD vs. CC. Researchers had previously confirmed that TRP was involved in phototransduction [28], energy balance control [26], and sensory functions [24]. In mammalian, the intrinsically photosensitive ganglion cells (ipRGCs) and melanopsin can be photoactivated, and then triggers G protein signaling pathway which can activate phospholipase C (PLC) and subsequent opening TRP [34]. As same time, TRP can be activated after light stimulus in *Drosophila* [35]. These studies indicated that TRP channel involved the light signal transduction in both vertebrates and invertebrates. So, when crabs were treated with different photoperiod, the expression level of TRP gene may be affected. However, the mechanism of how photoperiods affect TRP level is not clear. Some studies have proven that photoperiods can influence the Ca^2+^ concentration [36], and thereby influence the growth and survival of cells [37]. In crayfish, Ca^2+^ is related to photoreceptor sensitivity has been confirmed [15]. And TRP can be activated by Ca^2+^, to further participate in the process of phototransduction [38, 39]. In this study, we found that the expression level of TRP was changed by constant light or constant darkness. The above statement may provide an idea to explain how photoperiods affect TRP level. But the effects of TRP are on survival and growth in *E*. *sinensis* needs further research.

Additionally, we also found that guanine nucleotide–binding proteins (G proteins) gene had significant expression from the phototransduction pathway. G proteins which are made up of Gαq, Gβe and Gγe can be activated by the light rhodopsin [40–42]. As mentioned earlier, G protein can stimulate PLC activity and further mediated phototransduction[34, 42]. In fact, light signal transduction is a complex process, which can be regulated by a variety of substance interactions including G proteins, cGMP, and K^+^ exchanger [43]. In this study, the Gαq gene is significantly upregulated in the phototransduction pathway when crabs were placed in constant darkness seven days (Fig 6). Given this, we can predict that a condition of constant darkness may improve the Gαq expression in eyestalks and therefore influence the phototransduction pathway.

As mentioned previously, photoperiod can affect energy metabolism. We also found that the glycogenolysis and glycolysis pathways in *E*. *sinensis* eyestalk ganglion were significantly enriched for DEGs, and the PDC and PPC genes were involved in these pathways (Fig 7). PDC is an abbreviation for pyruvate dehydrogenase complex, as controlled by the pyruvate dehydrogenase kinase (PDK). It catalyses the oxidative decarboxylation of pyruvate to the acetyl coenzymeA (acetyl-CoA) which produced from the glycogenolysis and glycolysis pathways [44]. And acetyl-CoA plays an important role in the tricarboxylic acid (TCA) cycle which produce chemical energy in the form of ATP [45]. Therefore, PDC is indispensable in the metabolic process. On the other hand, researchers have found PDC E1 subunit deficiency in zebrafish visual mutants, and visual defect can affect energy metabolism [46]. Moreover, PDC subunits participate in severe neurological dysfunctions, including optokinetic response [47]. Meanwhile, in this study, we also found that photoperiods can affect PDC gene expression of *E*. *sinensis* eyestalk ganglion. Therefore, this study suggests that photoperiod possibly regulates the PDC activity by eyestalk ganglion, and then affects the energy metabolism process.

PPC is the only enzyme that can catalyze the conversion of oxaloacetate to the intermediate phosphoenolpyruvate in the glycogenolysis/glycolysis progress [48]. From our RNA-Seq results, we found the upregulation of PPC gene expression in the glycogenolysis/glycolysis KEGG pathway (Fig 8). In mammalian, hepatic glucose production is controlled by the gluconeogenic enzyme activity in the liver, PPC [49]. And PPC can be regulated by the forkhead transcription factor (Foxo1) which is a member of a highly-conserved DNA binding motif protein family and plays an important role in insulin signaling transduction [50]. There was reported that Foxo 1 mRNA level can be affected on different photoperiod and subsequent regulating PPC level in rats [32]. In a short, photoperiods regulated the PPC expression level which was possibly controlled by Foxo 1 expression. But, there was no research on the effect of photoperiod on PPC activity in crustaceans. This study provides new evidence for the effects of photoperiod on PPC gene expression in *E*. *sinensis*. In addition, we found that the pyruvate kinase (PK) gene was up-regulated in the glycogenolysis/glycolysis pathway. PK can catalyze phosphate group transfer and phosphoenolpyruvate conversion to adenosine diphosphate [51, 52]. PK not only takes part in carbohydrate metabolism [53–55] but is also involved in immunological reaction progress in crustaceans [56, 57]. A study on *Drosophila suzukii* found that photoperiod could regulate the PK levels, and then mediate the adult reproductive diapause [58]. Currently, there are few reports about the effects of different photoperiods on PK activity in crustaceans [59]. From our discoveries however, we found that different photoperiods regulate the expression of PK, and PK as an important enzyme, participates in energy metabolism. Therefore, photoperiod could affect energy metabolism progress though PK in crabs.

In the transcriptome, the oxidative phosphorylation pathway was significantly enriched in DEGs. The NADH peroxidase (NADH) and ATPase were included in this pathway (Fig 9). NADH, as a part of the pyridine nucleotide, can decompose hydrogen peroxide [60], which covers the cellular metabolism process [61]. For example, a study showed that temperature reduction could drop the oxidation matrix volume of NADH, and then decrease the oxidative phosphorylation capacity of mitochondria [62]. Another study also showed that radiation affected the oxidative phosphorylation process by oxidizing NADH to NAD^+^ [63]. However, NADH is important to mitochondrial metabolism in animals. There have been few reports about the effects of photoperiod on NADH activity [64]. In this study, constant light and darkness had upregulated the NADH peroxidase gene, which indicates that photoperiods affected metabolism activity of crabs. There was also a significant upregulation of ATPase gene activity in the LL and DD groups. ATPase is generated in the mitochondria and participates in oxidative phosphorylation in order to produce ATP [65, 66]. It is well known that ATP decomposition releases energy to promote cell growth, cell division and other vital movements [67]. Recently, research has shown that photoperiod significantly influences the Na^+^/K^+^-ATPase activity of gills of animals [68, 69]. The vacuolar H^+^- ATPase (V-ATPase) daily rhythm was also controlled by photoperiod in insects [70]. Together, these studies indicate that photoperiod may affect energy metabolism through affecting the activity of ATP enzyme.

In conclusion, photoperiod is important for animals, and man-made changes in photoperiods are bound to influence the physiological activity of animals. Among these physiological activities, the phototransduction and metabolism processes are significantly enriched in DEGs. Though RT-qPCR, we verified the upregulation of TRP in the phototransduction pathway and the downregulation of PDC and upregulation PPC in energy metabolism processes. Additionally, we also found that there is significantly increased gene expression of cGMP, PK, NADH peroxidase gene and ATPase genes in different pathways.

## Supporting information

S1 Fig GO annotation of DEGs in DD vs. CC. (ZIP)

S2 Fig GO annotation of DEGs in DD vs. LL. (ZIP)

S3 Fig GO annotation of DEGs in LL vs. CC. (ZIP)

S1 File Data. (ZIP)
